# *Helicobacter pylori *genotypes identified in gastric biopsy specimens from Jordanian patients

**DOI:** 10.1186/1471-230X-6-27

**Published:** 2006-10-04

**Authors:** Laila F Nimri, Ismail Matalka, Kamal Bani Hani, Marwa Ibrahim

**Affiliations:** 1 Department of Medical Laboratory Sciences, Jordan University of Science & Technology, Irbid, Jordan; 2 Pathology and Microbiology Jordan University of Science & Technology, Irbid, Jordan; 3 Surgery, Jordan University of Science & Technology, Irbid, Jordan

## Abstract

**Background:**

The genetic diversity of *Helicobacter pylori *can be analyzed at two different levels: the genomic variation between strains originating from different individuals, and the variation in bacterial populations within an individual host. We reported for the first time the *H. pylori *genotypes in Jordanian patients with gastrointestinal diseases.

**Methods:**

Upper endoscopy was performed on 250 patients with symptoms of gastrointestinal diseases. Multiple gastric biopsy specimens were taken from the antrum. All the biopsies were tested by PCR for the *H. pylori *virulence genes *vacA*, *cagA*, and *iceA*, and 151 were tested by histology.

**Results:**

The biopsies positive for *H. pylori *by PCR were 110/250 (44%), and by histology 117/151 (77.5%), and these results were highly associated (*P *< 0.02). Analyses of virulence genes revealed that *iceA2 *(73.6%) was the predominant genotype, the *vacA*s2 allele was more frequently identified than the *vacA*s1 allele, while the *cagA *genotype was low (26.4%). The presence of certain genotypes might be associated with each other, but the presence of certain genotypes was not significantly associated with the age, or gender of the patient.

**Conclusion:**

The results illustrate the geographic nature of the genetic diversity of *H. pylori*, as the identified genotypes are similar to those reported in neighboring countries. This study provides a baseline data of *H. pylori *genotypes identified in gastric biopsy specimens from Jordan, serving as a powerful epidemiological tool for prospective investigations to better understand the genetic diversity of this pathogen.

## Background

*Helicobacter pylori *is a gastric pathogen that chronically infects more than half of all people worldwide. In developing countries, 70–90% of the population carries *H. pylori*; almost all of these acquire the infection before the age of 10 years [1]. In developed countries, the prevalence is lower, ranging from 25 to 50% (8) [1], due to the improved socioeconomic conditions over the last few decades [2]. Therefore *H. pylori *infection in developing countries may contribute to childhood malnutrition and increase the risk or severity of infection by other gastrointestinal pathogens such as *Vibrio cholerae *[3]. Most infected individuals are asymptomatic or have chronic gastritis [1,4]. The differences in disease outcome may be the result of a number of factors that include; host factors, environmental factors, and differences in the prevalence or expression of bacterial virulence factors [4,5]. The genetic diversity of *H. pylori *can be analyzed at two different levels: the genomic variation between strains originating from different individuals, and the variation in bacterial populations within an individual host [6]. By using randomly amplified polymorphic DNA-PCR and DNA fingerprinting, it has been shown that strains from unrelated infected patients had unique finger prints, whereas strains isolated from family members had very similar although not identical patterns [7]. These results implied that differences observed between strains infecting individual family members occurred after primary infection. Such genetic diversity can be observed among *H. pylori *virulence genes; *cagA*, *vacA*, and *iceA*.

A vacuolating cytotoxin that injures epithelial cells is encoded by *vacA *gene [8,9], which contains at least two variable parts [10]. The *vacAs *region (which encodes the signal peptide) exists as s1 or s2 allelic types, among type s1 strains, subtypes s1a, s1b, and s1c have been identified [11]. The m (middle) region occurs as them1 or the m2 allelic type, among type m2, two subtypes have been identified, designated m2a and m2b. In general, type s1 m1 and type s1 m2 strains produce high and moderate levels of toxin, respectively, while s2 m2 strains show little or novacuolating toxin activity [10].

The *iceA *gene, encoding for a putative restriction enzyme, which appears to be induced when *H. pylori *encounters epithelial cells shows allelic variation according to point mutation, resulting in two allelic types, the *iceA1 *and *iceA2 *[6]. A study of *H. pylori *infection in patients subjected to an upper gastrointestinal endoscopy in Jordan reported high prevalence [12], and confirmed that its presence was significantly associated with gastritis and peptic ulcer. The current study reports for the first time in Jordan the *H. pylori *genotypes identified in gastric biopsy specimens.

## Methods

### Patients

A total of 250 consecutive patients who visited King Abdullah Hospital, and Princess Basma Hospital between July 2003 and May 2004, for upper endoscopy were enrolled in the study. These two teaching hospitals are affiliated with Jordan University of Science and Technology, where the study was conducted. Biopsy specimens were taken from the antrum. The study was approved by the Ethics Committee of the University. Each patient signed a written informed consent prior to specimen collection, and all clinical specimens were tested undercode.

### Data

The information provided in the pathology reports or patients' files was recorded for each patient, which included: patient's hospital number, age, gender, history, clinical diagnosis based on histology, endoscopy, and previous treatment (e.g., anti-*H. pylori*, three had proton pump inhibitors or antacids). The symptoms reported by the patients who underwent upper gastrointestinal endoscopy were abdominal pain, epigastric pain, vomiting, or heartburn.

### Histological examinations

Histological examination was performed on 151 (60.4%) antral biopsy specimens. Five specimens from the antrum mucosa were taken with medium-sized forceps. Two specimens were embedded in paraffin and the paraffin sections were stained using haematoxylin-eosin and Giemsa methods. The mucosal specimens were evaluated histologically according to the Sydney classification. Coded slides were examined microscopically by a single pathologist using a high power (magnification, ×400), and at least five high-power fields were examined.

### PCR-based genotyping of three virulence genes

All the 250 biopsies tested by PCR were stored at -80°C in 70% ethanol in eppendorf tubes until processed. These biopsies included the 151 biopsies that were tested by histology.

The biopsy specimens were homogenized with a sterile micro pestle, and DNA was extracted using Wizard Genomic DNA purification kit (Promega, Madison, WI, USA), following the manufacturer's instructions for the purification of DNA from animal tissue. The presence of *H. pylori *was detected by separate PCRs aimed at the *cagA*, *vacA *s and m regions and the *iceA *genotypes were determined by separate *iceA1*- and *iceA2*-specific PCRs as described previously [13,14]. Five species-specific primer sets (alpha DNA, Montreal, Canada) were used to amplify highly conserved regions within the indicated genes.

### Statistical analysis

The association between histology and PCR results, and the association between genotypes was analyzed using the Fisher's exact and chi-square tests statistical package for social sciences (SPSS Inc. Chicago, Illinois USA). The difference in mean age between males and females was calculated by independence sample t-test.

## Results

Diagnosis of *H. pylori *was based on histology, and PCR method.

### Histological findings

Histological examination of the 151 biopsies revealed that 117 (77.5%) patients were positive for *H. pylori*, therefore were actually infected, and 34 (22.5%) were negative.

### Virulence factors

The presence of the c*agA*, *vacA *s and m regions and the *iceA1*- and *iceA2 *genes were investigated in all the 250 biopsies. The biopsies that were PCR positive for one or more of the genes were 110 (44%), and 140 (56%) were negative for all genes.

The male: female ratio of the patient population was 58/110 (52.7%) males: 52/110 (47.3%) females; (mean age 42.03 + 15.135 years; range, 17 – 67 years).

### H. pylori genotypes

The results of genotyping in 110 biopsies are presented in Table 1.

#### vacA genotypes

The sizes of the amplified products for *vacA *s1 and *vacA *s2 are 259 bp and 286 bp respectively. The intensities of the products varied between specimens. The *vacA *s region was amplified in 75/110 (68.2%) biopsies. The s1 variant was detected in 34/75 (45.3%), or 34/110 (30.9%), compared to the s2 variant, which was detected in 41/75 (54.7%), or 41/110 (37.3%) of the biopsies. The *vacA *m region was detected in 47/110 (42.7%) biopsies. The m1 variant was detected in 23/47 (48.9%) or 23/110 (20.9%), compared to m2 variant detected in 24/47 (51%), or 24/110 (21.8%).

A combination of the *vacA *s, and m regions was detected in 26/110 (23.6%) of the biopsies. Both *vacA *s1m1 and *vacA *s2m2 were detected in approximately equal amounts in 12/26 (46.2%), of the *vacA *sm genotype, or 12/110 (10.9%), whereas *vacA *s1m2 was detected in only 2/26 (7.7%) of the *vacA *sm genotype, or 2/110 (1.8%). None of the biopsies showed the *vacA *s2m1 genotype, or multiple genotypes.

#### cagA genotype

The size of the *cagA *amplified product was 349 bp. The *cagA *genotype was detected in 29/110 (26.4%), and 81 (73.6%) were negative.

#### The association between the cagA and vacAs1 genotypes

The *cagA *genotype was detected in 8/17 (47.1%) of the *vacA *s1 genotype, compared to only 2/20 (10%) of the *vacA *s2 genotype.

#### iceA genotype

None of the 110 biopsies showed the *iceA*1 genotype, while 81 (73.6%) showed the *iceA2 *genotype. The *iceA2 *amplification yielded both the 229 bp and 334 bp fragments, this difference in the fragment size is due to the presence of a 105 bp in – frame amplicon present in the 334 bp fragment that is absent in the 229 bp fragment [15].

#### The association between the genotypes and gender

Although certain genotypes were detected more in one sex than the other, their presence was not significantly associated with the age, or gender of the patient. The genotypes that were detected more in males than females were the *vacA *s1 9/17 (53%), *cagA *16/29 (55.2%), *iceA2 *45/81 (55.5%), *vacA *s1m1 7/12 (58.3%), and the combined *vacA *s1 *cagA *genotypes 6/8 (75%). The genotypes that were detected more in females were the *vacA *s2 11/19 (65%), *vacA *m2 15/20 (75%), *vacA *s2m2 8/11 (72.7%), and the combined *vacA *s2 *cagA *genotypes 2/2 (100%). The *vacA *m1, and *vacA *s1m2 genotypes were detected in approximately equal amount in both sexes.

### Statistical analysis

The overall positive PCR results were highly associated (*P *< 0.02) with histology results. Analysis of data showed a significant association between the simultaneous detection of both *cagA *and *vacA*s1 genotypes (*P *= 0.026), the combination of both the *vacA*s1 and the *vacA*m1 genotypes (*P *< 0.0001), and the combination of both the *vacA*s2 and the *vacA*m2 genotypes (*P *< 0.0001). On the other hand, no association was observed between the detection of both the *vacA*s2 and *cagA *genotypes (*P *= 0.102), the detection of more than one genotype with either age or gender (*P *> 0.05), the combination of both the *vacA*s1 and the *vacA*m2 genotypes (*P *> 0.05), and the combination of both the *vacA*s2 and the *vacA*m1 genotypes (*P *> 0.05).

## Discussion

The present study reports on the *vacA*, *cagA*, and *iceA *genotypes of *H. pylori *that were identified in gastric biopsies. Although all strains carry a copy of the vacA gene, with either the s1 or s2 signal sequences, the *vacA *s region was amplified in 75/110 (68.2%) biopsies. Similar results were reported by other studies indicating that additional subfamilies of s and m genotypes beside the known ones may exist [16].

The predominant genotype in the 110 biopsies that were positive for *H. pylori *by PCR, was the *iceA2 *(73.6%), followed by the *vacAs *genotype (68.2%); 34 (45.3%) of these were the *vacA *s1 allele, and 41 (54.7%) were the *vacA *s2 allele, while the *cagA *genotype was amplified only in 29/110 (26.4%) of the biopsies. Our results are in agreement with other studies conducted on Israeli children [13], and Egyptian patients [15], where the *cagA *genotype was reported in 28%, and 36% respectively. The similarity of the genotypes identified in the three studies could be explained by a primary geographic influence important in the adaptation of the organism to the environment and climatic conditions [13], despite the obvious host differences in life style in two neighboring countries. The close resemblance of strains in neighboring countries was also reported in Bangladesh and Calcutta, India [3,17], which is quite likely considering the close proximity of the two countries, the similar physiological environments, and life styles of the host.

Higher prevalence (67% or more) of the *cagA *genotype in *H. pylori *was reported in Europe, Central and South America, and East Asia [15]. The *vacA *s2 allele was detected in less than 30% in the studied population in most of these countries. Prevalence rates of this genotype similar to the current study (54.7%) were reported in Egypt (50%) [15], while higher rates (65%) were reported in the Israeli study [11]. A study in Kuwait reported that *vacA *s1 and s2 types were detected in approximately equal numbers in biopsies obtained from patients of Middle-Eastern origin, while African Arabs were predominantly infected with the s2 type [18]. A study of genotypes in four different countries reported that the *cagA*, and *vacA *s1ml *iceA1 *genotypes were predominant in both Japan and Korea [14], and the *cagA*, *vacA *s1m1, *iceA2 *genotypes were frequently identified in the United States, while the *cagA*, *vacA *s1m1, *iceA2 *genotypes were predominant in Colombia. The same study reported higher prevalence of the *vacA *s1 than the *vacA *s2 genotype, and a high prevalence of the *cagA *genotype; however, the prevalence of the *iceA1 *and *iceA2 *genotypes varied among these countries. A study conducted in England reported that the *vacA *s1m1 genotype was found to be less common in England [19], while a predominance of *iceA1 *alleles, *cagA*, and the presence of *vacA *m1 alleles were observed. Turkish strains examined predominantly possessed the *cagA*, *vacA *s1m1, or *vacA *m2 genotypes, which were the typical genotypes in strains from Western countries [20]. The predominance of the *vacA *sl/m1 allelic combination, and a high prevalence of the *cagA *gene (87%) were also reported in Estonia *H. pylori *strains [21].

Based on the presence of a combination of the *vacA *s, and m alleles, the *vacA *s1 allele was significantly associated with *vacA *m1 (*P *< 0.0001), and the same association was observed between the *vacA *s2 and the *vacA *m2 (*P *< 0.0001) alleles. However, the detection of both *vacA *s1 and *vacA *m2 alleles was independent of each other (*P *> 0.05). The *vacA *s1m2 genotype was detected only in 2/26 (7.7%) of the *vacA *sm combination. In addition, there was no significant association between the *vacA *s2 and *vacA *m1, meaning that the detection of each allele was independent of the other (*P *> 0.05). This finding may explain the absence of the combined s2/m1 genotype from the isolates in the study that was reported previously [22,23]. However, the first case of *vacA *s2m1 *H. pylori *isolate was reported in a duodenal ulcer patient from South Africa [24].

The significant association between the *vacA *s1 allele and *cagA *genotype (47.1%) in our study was also reported in 50% of the Israeli and Egyptian isolates [13,14]. An association of more than 85% of the isolates was reported in other countries [15,22], confirming that the two markers are closely related. Our study showed no significant association in the detection of two genotypes in the same isolate such as the *cagA *with the *iceA*2, the *vacA *m2 allele with the *iceA*2 genotype, and the *vacA *s2, m2 alleles with the *iceA*2 indicating that the detection of one gene was independent of the other. Moreover, the detection of the combined *vacA *s1 m1, and *iceA*2 genotypes in few biopsies was insignificant (*P *> 0.05), indicating that the detection of *iceA*2 was independent of the *vacA *genotypes. The same findings were reported by a previous study [22]. None of the strains had multiple *vac*A genotypes, which were reported in other countries such as northern South America [15].

Females were more often carrying the *vacA *s2, and the *vacA *m2 genotypes (65%, and 75%, respectively) compared to (42%, and 25%) in males. The *iceA2 *(55.5%), *cagA *(55.2%), and *vacA *s1 (53%) genotypes were detected more in males than females. The gender of the patient and the detection of certain genotypes or combination of genotypes were not significantly associated. Moreover, the *H. pylori *genotypes (*P *> 0.05), and the detection of more than one genotype had no significant association with either age or gender of the patient.

The overall positive PCR results were highly associated (*P *< 0.02) with histology results. The differences in the histology, and PCR results could be due to the patchy distribution of the *H. pylori *in the stomach. Moreover, false negatives might be a problem in genotyping from biopsies since some biopsies were found to contain compounds inhibiting the PCR [25]. In addition, testing more multiple biopsies by histology compared to one by PCR increased the possibility of finding the bacterium, and explains the more positive results obtained by histology (77.5%), compared to PCR (44%). The treatments of patients with the proton pump inhibitors, antacids, or anti-*H. pylori *therapy may have lead to the negative results in the tests performed in these patients [26].

## Conclusion

Jordanian strains examined predominantly possessed the *iceA2 *allele, the *vacA*s2 allele was detected more than the *vacA *s1 allele, while the *cagA *genotype was low. The detection of certain genotypes might be associated with each other. The results illustrate the geographic nature of the genetic diversity of *H. pylori*, as the identified genotypes are similar to those reported in neighboring countries.

This study provides a baseline framework of *H. pylori *genotypes identified in gastric biopsy specimens, serving as a powerful epidemiological tool for prospective investigations to better understand the genetic diversity of this pathogen.

## Competing interests

The author(s) declare that they have no competing interests.

## Authors' contributions

All authors read and approved the final manuscript.

LN designed and coordinated the study, optimized PCR conditions, carried out molecular analysis and wrote the manuscript. IM performed histological examination, and interpretation of results. KBH recruited patients, carried out endoscopies and gastric biopsies. MF performed the PCR, participated in the data analysis.

## Pre-publication history

The pre-publication history for this paper can be accessed here:



## Figures and Tables

**Table 1:** Prevalence of *Helicobacter pylori *genotypes detected in 110 biopsies

**Genotype**	**Prevalence (%)**
*vacA*s1	34 (45.3)
*vacA*s2	41(54.7)
*vacA*m1	23 (48.9)
*vacA*m2	24 (51.1)
*vacA*s1m1	12 (46.2)
*vacA*s2m2	12 (46.2)
*vacA*s1m2	2 (7.7)
*vacA*s2m1	0 (0.0)
*cagA*	29 (26.4)
*iceA1*	0 (0.0)
*iceA2*	81 (73.6)
